# The relationship between utilization of mobile health technology and in-person care

**DOI:** 10.1186/1940-0640-10-S1-A13

**Published:** 2015-02-20

**Authors:** Joseph Glass

**Affiliations:** 1 Department of Social Work, University of Wisconsin-Madison, Madison, WI, 53706, USA

## Background

Persons with alcohol use disorders benefit from continuing care, but few receive aftercare or ongoing monitoring after completing an episode of addiction treatment. A recent randomized controlled trial (RCT) demonstrated that a smartphone intervention provided effective continuing care for alcohol use disorders. The Addiction-Comprehensive Health Enhancement Support System (A-CHESS), which provides relapse prevention services including psychoeducation, communication, support, and electronic monitoring, significantly reduced risky drinking days and increased abstinence over 12 months following discharge from residential treatment. While smartphone interventions appear to be an effective mechanism for delivering continuing care, it may also be of interest to harness this technology to facilitate engagement in formal or “traditional” face-to-face treatment for addiction. We sought to evaluate the feasibility of using smartphone interventions to facilitate engagement in formal continuing care for alcohol use disorders.

## Methods

We conducted secondary analyses of the A-CHESS open-label RCT, which recruited 349 patients with alcohol use disorders from residential treatment programs. Participants were randomized to A-CHESS plus treatment as usual or treatment as usual at discharge. In semi-structured interviews, an adapted version of the Treatment Services Review assessed outpatient and residential addiction treatment utilization at baseline, 4, 6, and 12 months. We described outpatient and residential addiction treatment utilization at each interview and used chi-square tests to evaluate differences in treatment engagement between A-CHESS recipients and controls.

## Results

Participants were 80 percent white, 39 percent female, and had an average age of 38 years. Most participants (62.5%) had comorbid drug use. Participants who received the A-CHESS intervention had higher rates of outpatient treatment attendance at each follow-up interview compared to controls, with significant differences observed at 8-month (29.5% vs. 18.5%; X^2^[1, N = 297] = 4.856; p = 0.030) and 12-month (25.7% vs. 14.5%; X^2^ [1, N = 281] = 5.6; p = 0.025) follow-ups. Differences in return to residential treatment were not statistically significant at any follow-up.

## Conclusions

Smartphone interventions hold promise as both a mechanism for providing continuing care and promoting engagement in formal outpatient treatment for alcohol use disorders. Future research should evaluate ways to use smartphones to reach out to more segments of the population of persons with alcohol use disorders with unmet treatment needs.

## Trial registration

ClinicalTrials.gov NCT01003119

ClinicalTrials.gov NCT01963234

## Grant support

NIAAA 5R01AA017192-05

NIDA 5R01DA034279-03

